# Ranking ecosystem services delivered by trees in urban and rural areas

**DOI:** 10.1007/s13280-022-01722-2

**Published:** 2022-03-28

**Authors:** Patrycja Przewoźna, Krzysztof Mączka, Marcin Mielewczyk, Adam Inglot, Piotr Matczak

**Affiliations:** 1 Faculty of Geographical and Geological Sciences, Adam Mickiewicz University, Bogumiła Krygowskiego 10, 61-680 Poznań, Poland; 2 Faculty of Sociology, Adam Mickiewicz University in Poznań, ul. Szamarzewskiego 89c, 60-568 Poznań, Poland; 3 Institute of Anthropology and Ethnology, Faculty of Anthropology and Cultural Science, Adam Mickiewicz University in Poznań, ul. Szamarzewskiego 89, 60-568 Poznań, Poland; 4 Faculty of Civil and Environmental Engineering, Gdańsk University of Technology, ul. Narutowicza 11/12, 80-233 Gdansk, Poland

**Keywords:** Analytic hierarchy process, Ecosystem services, Environmental management, Expert knowledge, Tree protection

## Abstract

Policies and strategies for tree management and protection on a national, regional, and local level have not sufficiently considered differences between rural and urban areas. We used expert knowledge to compare rural and urban areas in a case study evaluating the relative importance of ecosystem services (ES) in policy development. The Analytic Hierarchy Process (AHP) and focus group discussions were used to rank 17 ES, representing four classes of services: provisioning, regulating, habitat, and cultural. The results indicated that effective protection strategies, beyond simply increasing general tree cover, should consider specific benefits trees provide to local communities. We discuss the role of objective prioritization of ES delivered by trees in urban and rural areas and their consequences for decision-making processes.

**Supplementary Information:**

The online version contains supplementary material available at 10.1007/s13280-022-01722-2.

## Introduction

Tree canopy cover is diminishing across the globe (Hansen et al. 2013). Losses occur primarily due to increasing urbanization (Morgenroth et al. 2017; Nowak and Greenfield 2020) and the expansion of agricultural land (Nowosad et al. 2019). Thus, effective tree management practices are becoming crucial in both urban and rural settings, especially since green spaces are vital to human quality of life and well-being (Díaz et al. 2015).

Decision-making for tree management is usually a complex issue. That is why Hayati et al. (2013) proposed a decision-making process that had three main components: criteria selection, setting the relative importance of criteria and a spatial multi-criteria evaluation. Spatial multi-criteria evaluation is facilitated by methods based on Geographic Information Systems (Booth et al., 2017), which offer advanced possibilities for data processing. However, the selection and prioritization of criteria used for decision-making can be ambiguous. They involve inherent trade-offs between socio-political, environmental, and economic costs and benefits. Assessing them is often complicated by differences in stakeholder opinions (Huang et al. 2011). As a result, tree management is not merely an attempt to increase tree coverage, but requires narrowing into operational actions, taking into account the needs and demands of local stakeholders; their views on the importance of trees (e.g., improving human health and esthetics and contributing to biodiversity conservation); and the limited budgets available for managing green spaces. This paper deals with the component of environmental decision-making that involves establishing the relative importance of different ecosystem services (ES).

This problem is discussed in the context of ES delivered by trees, which for the most part are currently not considered in decision-making related to tree management (“The ecosystem services concept in tree management” section). Meanwhile, the effectiveness of tree conservation strategies has direct effects on the benefits provided by trees (“Relative importance of ES delivered by trees” section), and whose importance can vary significantly between rural and urban areas because of socio-economic differences that can vary with geographic location (“Trees in urban and rural areas” section). The lack of unified standards for assessing the significance of ES provided by trees while taking into account location is a challenge addressed in the current study. Two main goals were identified: (1) To propose a methodology for ranking ES, and (2) To assess the importance of individual ES provided by trees in a case study of differences between urban and rural areas. The methodological approach applied is discussed in detail in subsection 1.4. We show how it enabled us to decrease the complexity of ES classification to its most parsimonious form. The final ES rankings that were obtained offer insights useful in tree management in rural and urban municipalities and the methods used are applicable to different types of areas.

### The ecosystem services concept in tree management

The concept of ES is increasingly acknowledged as a useful tool to support decision-makers (DMs) in environmental management. ES are understood to be all the benefits that humans receive from the natural environment. Although the publication of the Millennium Ecosystem Assessment (MEA 2005) popularized the ES concept (Hasse et al. 2014; Krajter Ostoić and Konijnendijk van den Bosch 2015; Brocker et al. 2017; Raum et al. 2019), it has had limited impact on environmental policies and practices at the national, regional, and local level (Schröter et al. 2016; Beaumont et al. 2017; Raum et al. 2019).

A review of scientific literature on urban forestry (Krajter Ostoić and Konijnendijk van den Bosch 2015) showed a worldwide increase in interest in the quantification of ES, especially carbon sequestration. However, cultural ecosystem services (CES) related to non-material ecosystem benefits are less often evaluated than benefits related to natural processes and wood provision (Brockerhoff et al. 2017). This is mainly attributable to the availability of information needed for such analyses. Remote sensing is one of the most easily accessible sources of data on trees. The i-Tree method (https://www.itreetools.org/) enables using the data to provide information about some ES delivered by them (such as carbon sequestration, oxygen production, and run-off retention). Nevertheless, in practice, it has been found that data availability alone is insufficient to prompt improved policies and practices concerning trees (Raum et al. 2019).

When i-TreeEco was applied in Great Britain (Raum et al. 2019), it was found to be an effective tool to raise public awareness and enhance ES assessment (see also Haase et al. 2014). However, information about ES delivered by trees obtained with i-TreeEco has rarely been used to develop tree-related policies and management practices. Other reviews verifying the practical applications of ES assessment also support this observation (Schröter et al. 2016; Beaumont et al. 2017).

### Relative importance of ES delivered by trees

There are many ES delivered by trees (Kronenberg 2012) as providing wood and fruits or regulating environmental processes (e.g., purifying the air and offering shade). Trees also provide habitat for plants, animals, and humans, as well as cultural benefits, such as increasing the esthetic value of landscapes and delivering recreation ecosystem services (RES), which is the most common type of CES (i.e., Ali et al. 2020; Jang-Hwan et al. 2020). Trees provide multiple ES simultaneously, but not always to the same extent. One of the main factors determining the types of ES provided is tree species (Davies et al. 2017; Aronson et al. 2017; Felton et al. 2020; Pretzsch et al. 2021). For example, Scots pine (*Pinus sylvestris*) is more valuable than Norway spruce (*Picea abies*) for many ES, including esthetics and recreation (Felton et al. 2020). Nowak and Aevermann (2019) also underline the importance of tree size since it strongly affects many ES, such as pollution removal and protection from the sun. They propose calculating the loss of future values that would occur if trees were removed, and suggest that compensation for tree removal be based on the ES losses. This approach may increase the effectiveness of environmental management, since it accounts for some ES provided by trees (although the method does not account for either CES or provisioning services).

Green space composition is another key factor influencing ES services, especially in urban areas (Aronson et al. 2017; Davies et al. 2017). If preserving biodiversity is most important, then spatial planning should protect and develop heterogeneous green spaces (Aronson et al. 2017). Furthermore, while a single tree planting may be of social significance locally, beyond providing some CES, its environmental benefits are minor (Davies et al. 2017), especially when the tree is young. Tree age is another important aspect of green space composition, for instance in the case of cooling provided by trees, the benefit is usually much greater when trees are older than 50 years (Pretzsch et al. 2021).

Knowing which ES are most important to the local community is crucial for effective tree protection strategies, and objective criteria should dictate decision-making in this regard. Decision-making should account for both the biological value of trees and their social significance. Due to the variety of measurement methods and ways of expressing the benefits of different ES, comparisons of ES are optimally done by ranking the importance of the benefits that trees provide. The most significant constraint of previous research on ranking ES, no matter the method applied (Wagner et al. 2019; Ali et al. 2020; Jang-Hwan et al. 2020) is caused by limitations in the number of ES analyzed. In addition, the choice of ES evaluated was often dictated by data availability and not necessarily by the importance of ES in specific locations. For this reason, many studies examine regulating ES, which can be relatively easily measured using remote sensing data and the i-TreeEco model (Raum et al. 2019). In comparison, some ES, such as those related to forest biodiversity, require large amounts of information and for that reason have only recently begun to attract interest from researchers (Brockerhoff et al. 2017).

### Trees in urban and rural areas

The importance of ES can differ between rural and urban areas. For example, the problem of deforestation occurs in both types of landscape (Nowosad et al. 2019), but societal perception of ES in each location can differ (Suchocka et al. 2019; Ali et al. 2020). Another example was shown for ES delivered by rivers in the Hexi Corridor Region in China, where farmland irrigation was the most crucial biotic ES for rural residents and RES were much less important, although RES are the most valuable type of services to people in urban areas (Ali et al. 2020). A similar differentiation based on urban–rural divisions can be observed for ES delivered by trees, especially given differences in the perception of trees by urban and rural populations (Suchocka et al. 2019).

ES rankings are usually carried out in case studies, which can make comparisons of different areas difficult. For example, when Wagner et al. (2019) reported the importance of common tree species in coffee-agroforestry systems in Tanzania, their rankings focused on selected provisioning and regulating ES, based on interviews with farmers who were asked to independently identify ES crucial to them. Thus, although farmers theoretically could have chosen any ES, some services were likely omitted because respondents were unaware of their existence, not because of their low importance. Providing respondents with a predefined list of ES would have allowed an unambiguous interpretation of results, although in practice, it can be challenging to consider all of the numerous ES delivered by trees (Kronenberg 2012). For that reason, Jang-Hwan et al. (2020), who evaluated the relative importance of seven ES provided by trees in urban national and neighborhood parks in South Korea that represented four ES classes, chose only recreation as an example of CES. In the case studied by Jang-Hwan et al. (2020), RES indicated by residents were the least important of all compared services. However, it is possible that a different result would have been obtained if a different ES had been used to represent particular classes of ES.

These are examples of the types of uncertainties that can occur when there is no predefined set of ES applied in research, or when the number of ES analyzed is limited. For this reason, establishing the relative importance of ES delivered by trees is crucial. Ranking all ES, at least as a first step, potentially optimizes environmental management and eliminates uncertainties caused by arbitrary selection of ES. In the case of ES in urban and rural areas, there has been no reliable prioritization of ES that could provide at least preliminary guidelines for tree protection strategies in each type of municipality. While differences in ES rankings between urban and rural areas are not the only factor that should be considered for tree management, it is by far the most important factor to begin with.

### Ranking ES with the AHP method

Analytical Hierarchy Process (AHP) is a method enabling both prioritization of compared criteria and establishing their relative importance (Saaty 1980), which has found wide application in environmental decision-making (Schmoldt et al. 2001). It has been used, for example, in sustainable forest management (Maroto et al. 2013; Uhde et al. 2015), for prioritizing a framework for managing invasive alien plants (Potgieter et al. 2018) for zoning areas of environmental fragility (França et al. 2019), and to evaluate ES delivered by urban parks (Jang-Hwan et al. 2020).

Prioritization with AHP can be performed by DMs, comparing elements in pairs using Saaty's (1980) 9-point fundamental scale of preferences (Table 1). This scale enables DMs to express their experience and knowledge in the form of a comparative number indicating by how much one element is deemed more important than another. This numerical evaluation in pairs is a significant advantage of AHP over other ranking methods, as it allows the DM to account for even the most minor details related to the comparison. From a psychological point of view, pairwise comparisons are also more natural than a combined evaluation of all objects at once (Prusak and Stefanów 2014). These are significant advantages when a large number of elements are compared as is frequently the case when ranking ES provided by trees. In addition, the AHP method allows the importance of the elements that are compared to be assigned weights, so it is possible to determine not only which are less and which are more important but also by how much, which provides additional interpretative power.

**Table 1:** Pairwise comparison scale used in AHP

Intensity of importance	Explanation
1	Both ES are equally important
3	Indicated ES is slightly more important than another
5	Indicated ES is strongly more important than another
7	Indicated ES is very strongly more important than another
9	Indicated ES is extremely more important than another – the highest possible order of affirmation
2, 4, 6, 8	Intermediate values

Pairwise comparisons may be done by several DMs together during a discussion session (e.g., Potgieter et al. 2018; Jang-Hwan et al. 2020) or individually. When comparisons are done individually, the Aggregation of Individual Priorities (AIP) is done using a geometric mean of all pairwise comparisons (i.e., Maroto et al. 2013; Trivedi and Singh 2017; Nyimbili and Erden 2020). When comparisons are made in a discussion session the most problematic issue is the time-consuming process of considering many pairs of factors. Using AIP solves this problem and also eliminates the risk that the final results would be more reflective of the opinions of the strongest personalities in the group (Prusak and Stefanów 2014). On the other hand, discussion reduces the likelihood of biased judgments, which may occur when DMs compare factors independently (Ishizaka and Labib 2011).

We decided to apply the AHP method combining both approaches. Since there were many ES to be compared, we used AIP. However, discussion sessions were also used to enable the exchange of opinions concerning pairwise comparisons for ES where the most significant disagreements occurred. Those comparisons are crucial, since they are characterized by high dispersion of individual judgments, which are difficult to express in a single number (Regan et al. 2006; Jaskowski et al. 2010). This procedure enabled the ranking of 17 ES delivered by trees, indicating key differences and similarities between two case studies.

## Materials and methods

### Locations used in the case study

**Fig. 1:**
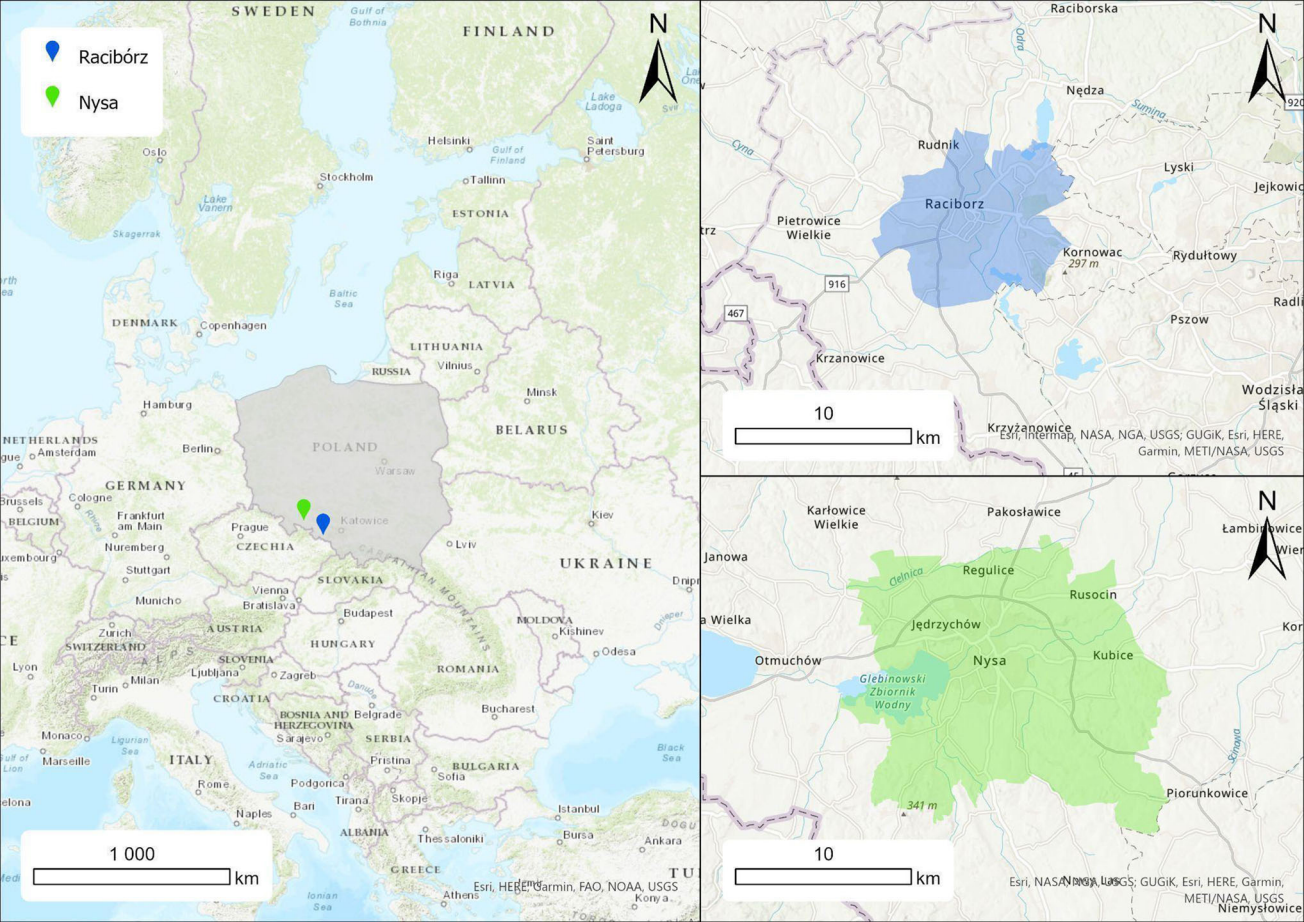
Location of case study

This study examines the relative importance of ES in Poland, one of the Baltic countries. The areas selected for analysis were a medium-sized city—Racibórz, and the rural part of the municipality of Nysa, located in south-western Poland, near the border with the Czech Republic (Fig. 1). Municipalities were comparable in land area, population size, and regional characteristics, including the presence of predominantly agricultural landscapes with little forest cover in the surrounding land. These municipalities are relatively typical for Poland based on socio-demographic and environmental indicators.^1^

The choice of two case study areas, one urban and one rural, allowed us to test the methodology developed for ranking ES and to assess the importance of individual ES provided by trees in two different social contexts.

### Procedure of ranking ES

**Fig. 2:**
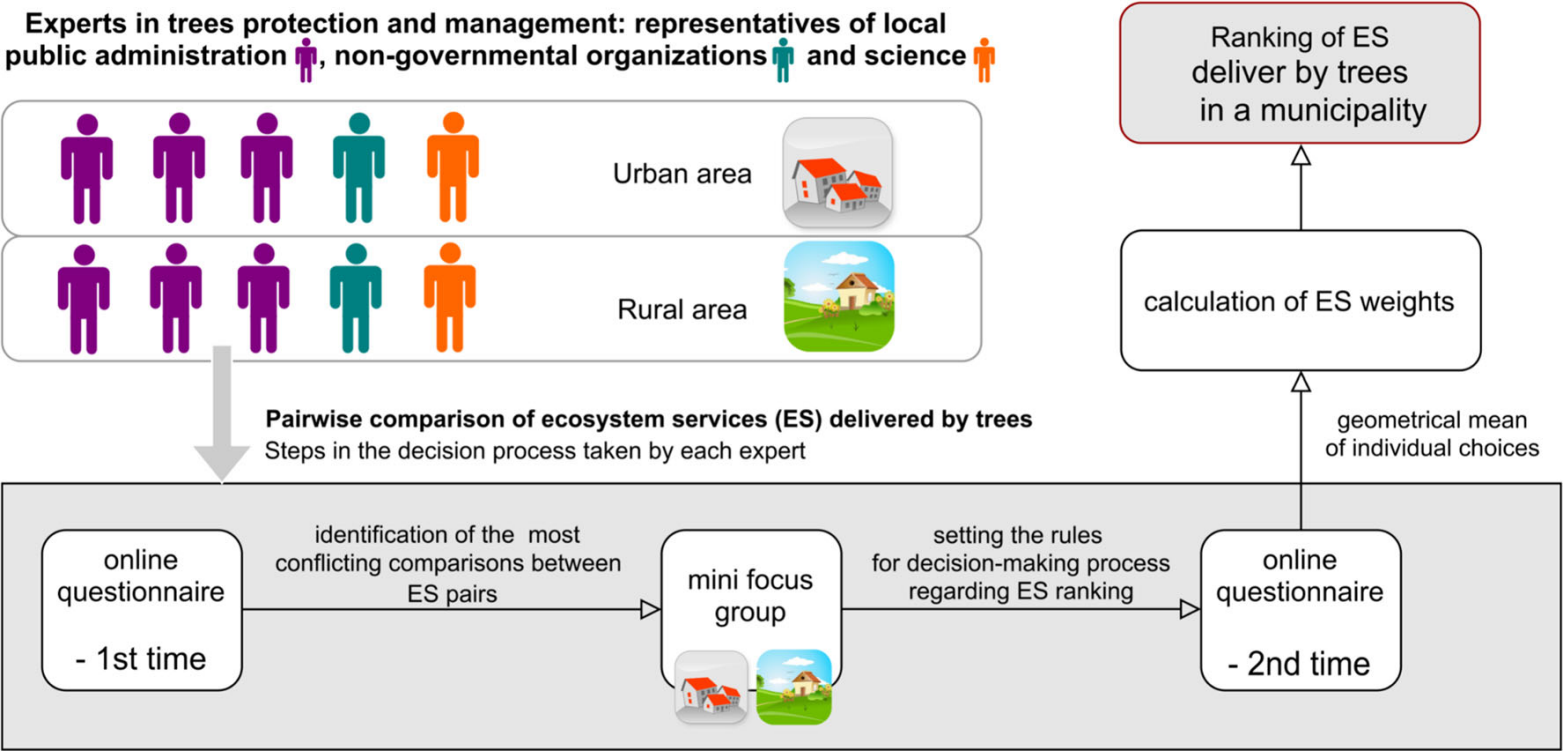
Procedure for ranking ES based on expert knowledge using the AHP method

Rankings of ES were made by ten experts invited due to their competencies in tree management, with two equal groups: one group selected for the urban area and the second for the rural area. The ranking procedure consisted of three steps: (1) completion of a questionnaire ranking ES using the AHP method, (2) discussion between experts in mini focus groups, and (3) completion of the same questionnaire a second time following discussion, to review previous rankings (Fig. 2). Each group was limited to five participants, as a larger group would have significantly increased discussion time. To obtain ES rankings that represent as wide a variety of existing opinions as possible, we identified high-profile experts with large diversity of experience and competence. Within each group we invited participation by three representatives of public administration (two managing the local Environment Conservation Department and one representing the National Forest Holding), a representative of a local NGO working in environmental protection, and an environmental scientist. We also made sure that, as far as possible, participants in both groups were diverse in age and gender. In this way, we aimed to minimize the impact of differences in expertise and background on ES rankings.

**Fig. 3:**
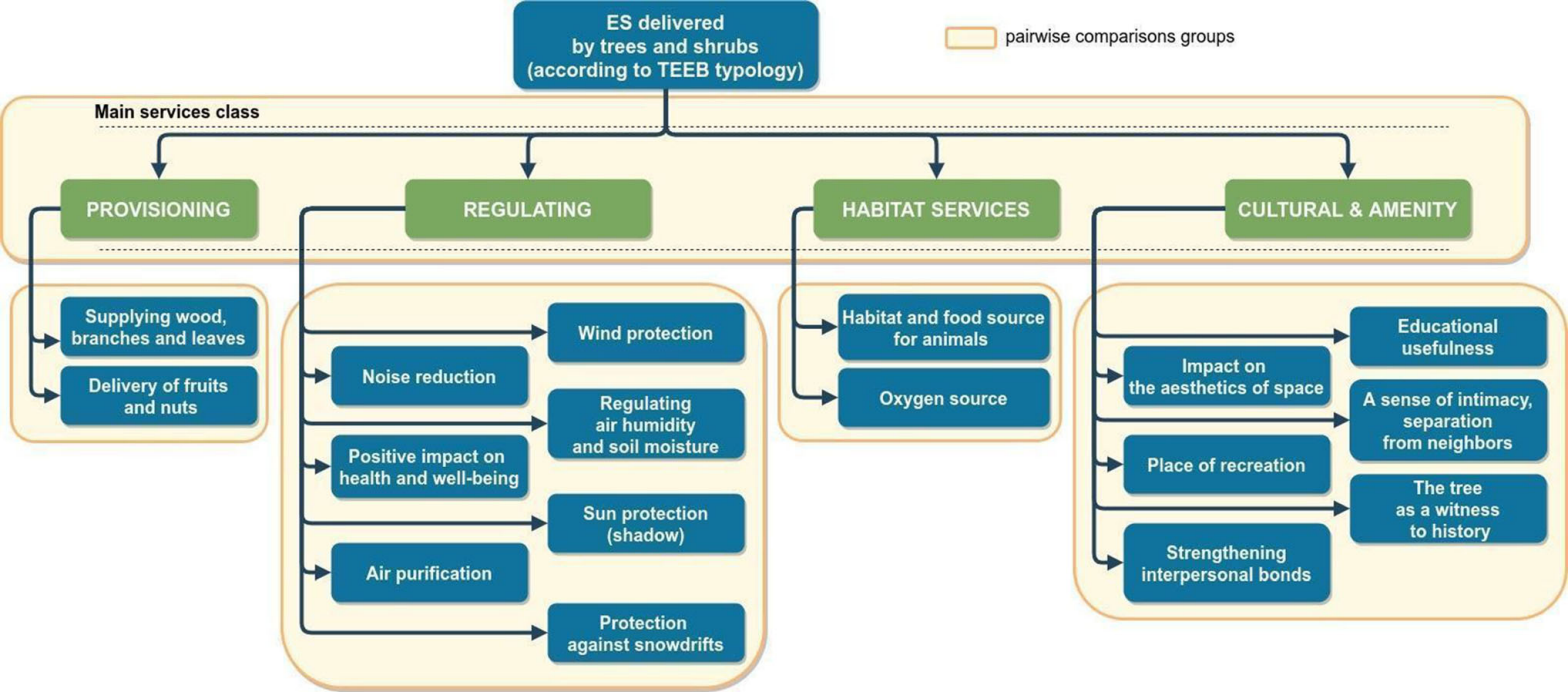
The hierarchy of ecosystem services used in pairwise comparisons

When the AHP method is used to solve multi-factor complex problems, a hierarchical structure needs to be created to reduce cognitive errors that can occur when a large number of comparisons are made (Saaty 1980). For the comparisons in this study, we prepared a list of ES on The Economics of Ecosystems and Biodiversity (TEEB 2010) classification of ES and its adaptation for trees in Poland by Kronenberg (2012), which we modified as follows before application in our research. Firstly, scientific jargon describing the list of ES was minimized to improve comprehension by non-scientists. Then we conducted a pilot study based on 10 semi-structured in-depth interviews (IDI) with farmers, city dwellers, students, etc. The goal was to establish a list of ES understandable to respondents, and to unambiguously name ES offered by trees. During the pilot study we wanted to find out which green spaces respondents use, whether they are home gardens, city parks or state forests, how they use these areas, what types of activities they perform, and what ES provided by trees and shrubs they use. We challenge the categories proposed by Kronenberg (2012) based on feedback from our respondents. First, as judgment of ES can depend on the perspective of the respondent, we sought to establish whether ES were favorable or unfavorable, and if so, for whom? For example, respondents could interpret the "place of animal life" in different ways, as in the case of beehives, which can be interpreted either as something positive (pollination) or negative (danger). Secondly, we addressed the difficulties distinguishing similarly named types of ES created by Kronenberg such as "water retention", "humidification of air and soil" and "creating areas of coolness and humidity". Thirdly, difficulties understanding some ES types were identified, where interviewees themselves pointed out instances of lack of clarity and asked for explanations of their meaning. Finally, after adjustments, we came up with a hierarchical list of 17 ES that were to be considered in pairwise comparisons as shown in Fig. 3. Five comparison groups were created – four with individual ES representing different ES types and one that rank the four main ES classes (i.e., provisioning, regulating, habitat, and cultural). The final pairwise comparison was made in the form of a matrix, and its eigenvector (denoting the relative importance of each factor) was calculated. In this way, the local weights of ES were compared and the main classes of service types evaluated. Then the rankings of all services provided by trees were obtained from the global weight of each ES, calculated by multiplying its local weight by the local weight of the main service class to which it belongs. A similar approach was used by Kil et al. (2016) and Potgieter et al. (2018).

The pairwise comparison of criteria and sub-criteria can be inconsistent due to distractions during the ranking process. Thus, a consistency ratio (CR) was calculated for each comparison matrix, determining how much the results obtained differ from purely random values, which indicates their reliability. When CR ≤ 0.1, the matrix is considered consistent (Saaty 1980). As the research had to be carried out online due to the COVID19 pandemic, a user-friendly application was developed and tested. It enabled easy comparison of ES and verified the CR for answers by each expert, ensuring the internal consistency.

Initially, participants completed an online questionnaire that enabled us to identify the seven most conflicting pairs of ES, using the degree of disagreement scale (Appendix S1). These pairs were discussed in two mini focus groups (Greenbaum 1998; Edmunds 1999) following the Utrecht method (Bolt et al. 2015; Shawahna 2018). Both focus group sessions lasted four hours and were conducted online via Microsoft Teams software in April 2021. The discussions aimed to broaden the mutual knowledge of experts and acquainted them with alternative viewpoints, but were not necessarily intended to achieve compromise (see the scenario with a complete questions list in Appendix S2). At the end of the discussion, experts provided general criteria that DMs should consider when ranking ES. They were asked to follow those criteria when verifying their decisions about the importance of ES delivered by trees.

Since the unequal distribution of ES among service classes in the hierarchical structure of ES may influence the results (Omamalin et al. 2014), this issue was discussed in detail in mini focus groups. Participants were familiar with the consequences of hierarchical structure on the final results. They were also asked to minimize it if needed by assigning more importance to those classes which apply to more ES, when verifying their rankings. After discussion, experts were requested to fill out the same questionnaire again. Based on the results of their second completed questionnaire, global weights were calculated, and the final ranking of ES delivered by trees in urban and rural areas was established.

Global weights were also calculated separately for each expert, enabling assessment of the experts' self-agreement before and after the discussion session. Non-parametric statistical analysis was done using Kendall's W statistic. In addition, a change in the degree of consent for each pairwise comparison was also evaluated. We counted the number of cases in which there was an apparent disagreement among experts about which ES should be considered more critical. The influence of discussion was measured with an odds ratio (OR). All statistics were carried out using R-software.

## Results

**Fig. 4:**
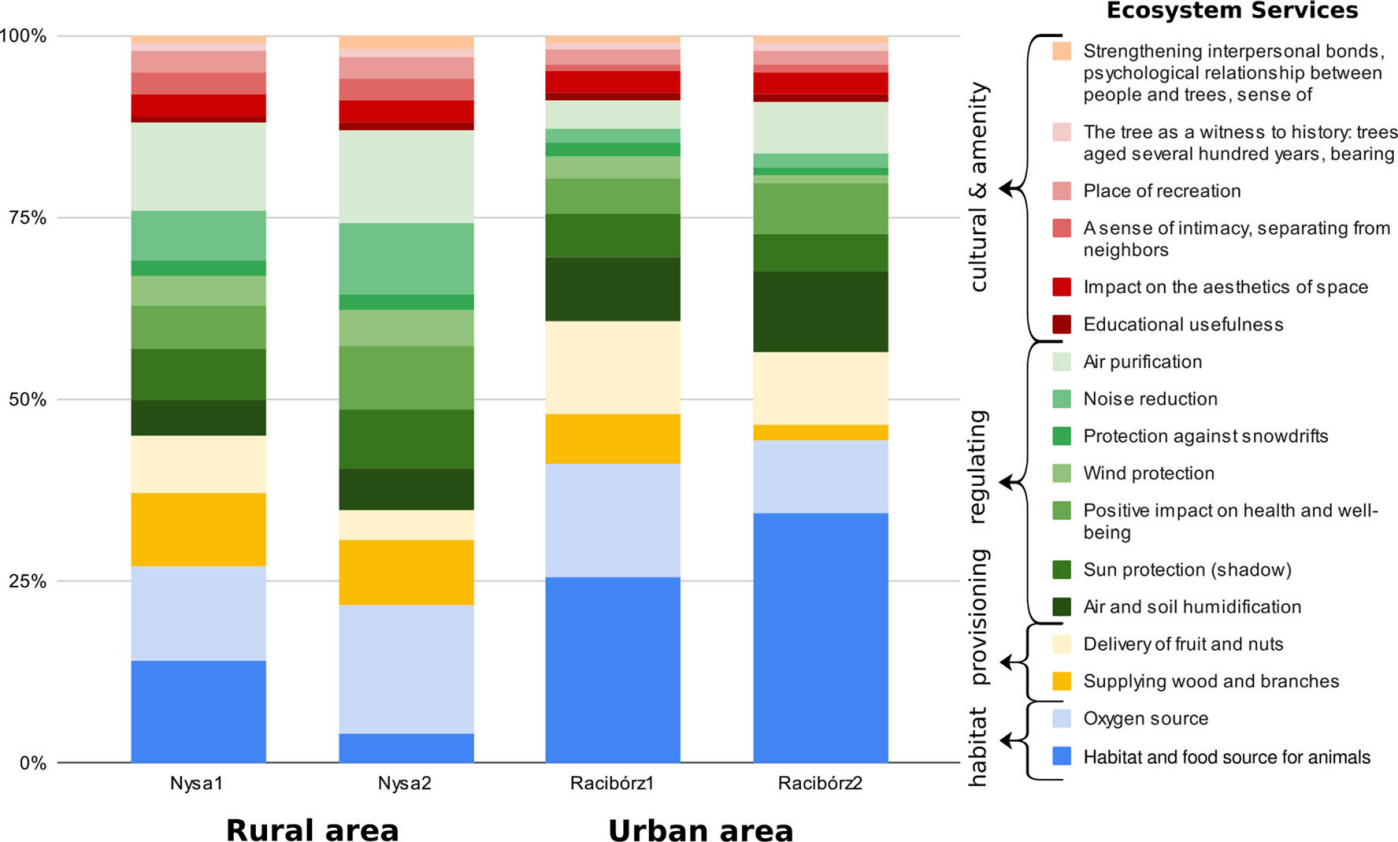
Importance rankings obtained by the AHP method for ES provided by trees in each study area before (1) and after (2) the discussion in focus groups

There were significant differences in rankings of the importance of ES between experts from rural Nysa and those from urban Racibórz. These differences were noticeable when comparing classes of services delivered by trees and for each individual ES, both for the first response to the online AHP questionnaire as well as the second response (Fig. 4). In both locations, the most important ES classes, indicated both before and after discussion, were related to providing habitat services and regulating environmental processes. In Racibórz, there was only a slight difference in assigned weights between these ES classes, but habitat services were considered more important. In the rural part of Nysa, regulating services were considered more important in the first response to the questionnaire, a result that was strengthened by discussion among participants. Provisioning services and CES were rated much less important by experts from both study areas, both before and after discussion. Focus group discussions had a notable influence on ES rankings. When redoing the questionnaire after a discussion session, experts from both locations increased their ranking of the importance of regulating services, as shown by increased importance in the rankings of individual ES. In contrast, focus groups only slightly increased the importance of CES and of several of the individual ES in this class. In general, group discussions increased agreement among experts.

**Fig. 5:**
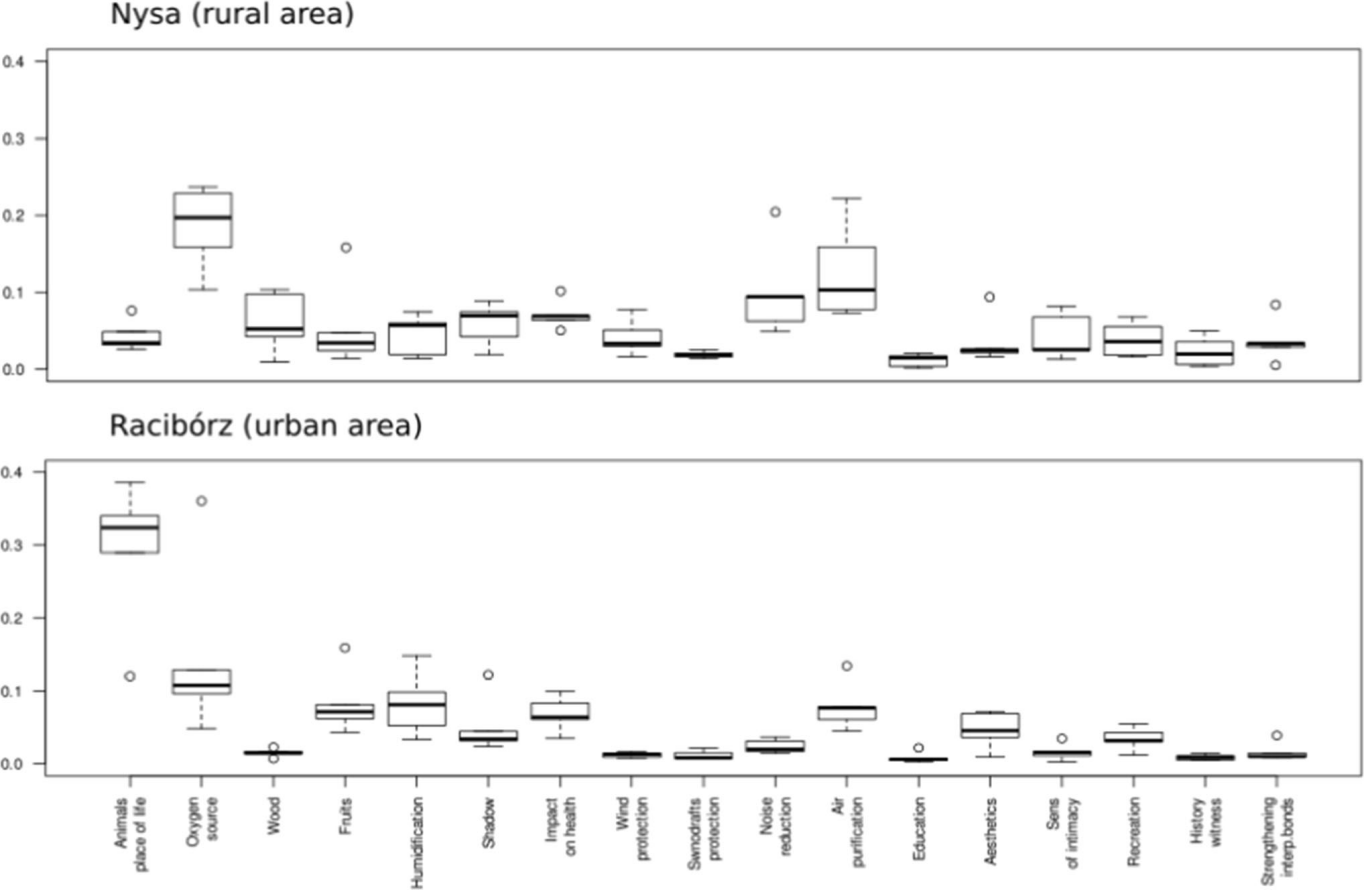
Global weights assigned to each ES calculated for each expert from Nysa and Racibórz after the second filling of the online questionnaire

Global weights for ES rankings calculated based on each expert’s responses were at least moderately consistent before and after discussion sessions. However, discussion increased agreement for both the urban and rural groups (Fig. 5). A greater increase in agreement occurred in the urban group, increasing from moderate agreement (*W* = 0.54, *p* < 0.001) to near unanimity (*W* = 0.81, *p* < 0.001). Agreement in ES rankings among experts in rural Nysa increased to a lesser extent between the first (*W* = 0.47, *p* < 0.01) and the second response to the online questionnaire (*W* = 0.58, *p* < 0.001).

**Fig. 6:**
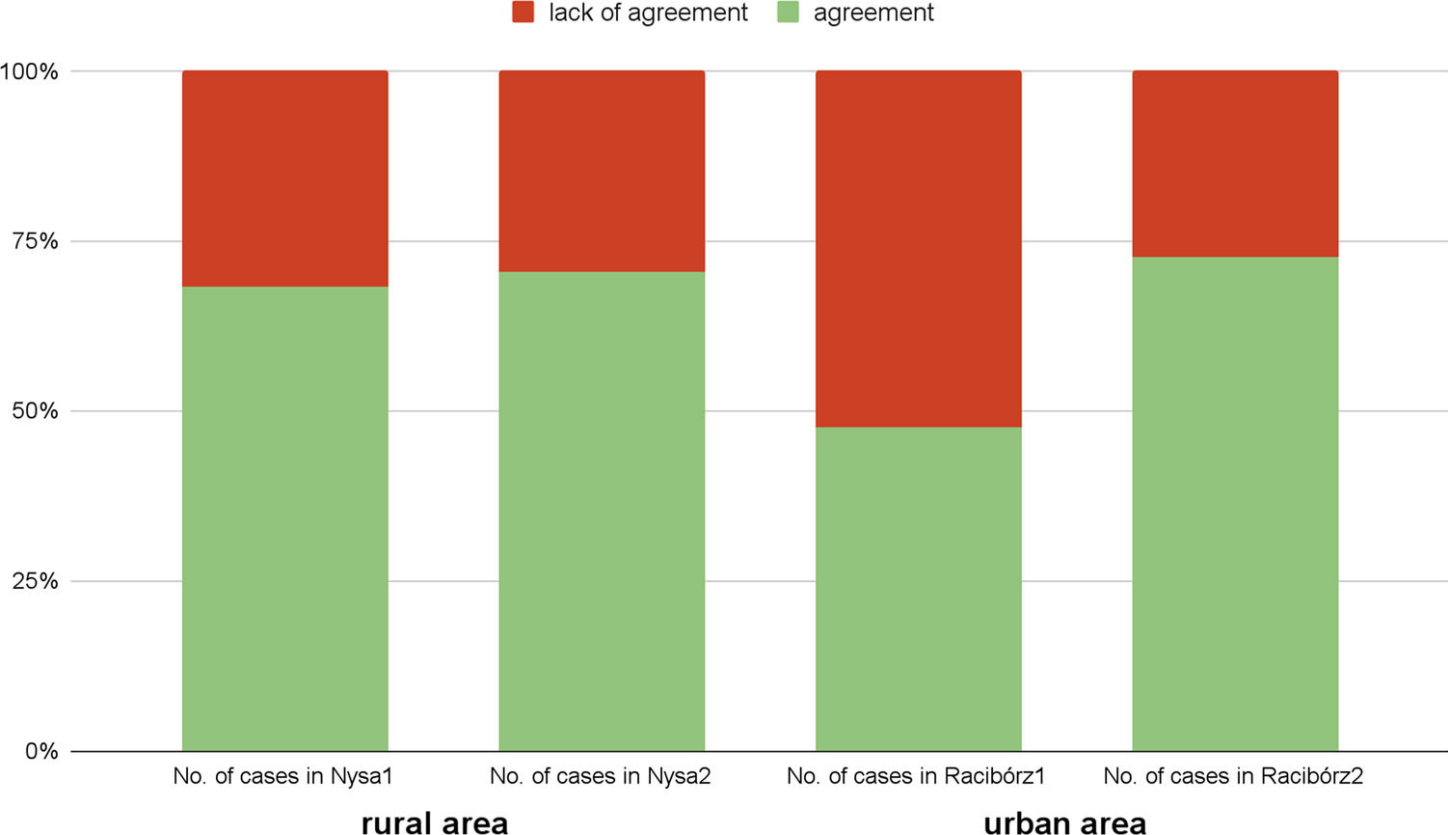
Share of pairs representing agreement before (1) and after discussion (2) in each case study location. Detailed comparisons expressed in a ten-degree of consent scale are described in Appendix S1

The same effect was observed when we evaluated the degree of consent of individual pairwise comparisons (Fig. 6). In the case of Racibórz, a larger number of pairs lacked agreement, occurring in 53% of cases; after discussion, there were disagreements about only 27% of pairwise comparisons. According to odds ratio statistics, the chance of a lack of agreement was 2.88 times greater without discussion (significant difference within the 95% confidence interval: 1.10–7.86). For respondents from Nysa, the likelihood of agreement was the same with or without discussion (OR = 1.11, 95%, 0.41–3.05). However, this could be attributed to rural experts having a higher initial level of agreement (Table 1).

Following mini focus groups, there were changes in relative importance assigned to ES delivered by trees, even for pairs of ES not discussed during the online meeting. In Racibórz, experts identified the following criteria that DMs should account for when ranking ES delivered by trees: (1) the need for universal availability of ES, (2) local context related to the city ecosystem (i.e., environmental conditions, pollution), (3) the importance of selected benefits delivered by trees for the local community, (4) the priority of ES already available in an area over those that could be provided in future, and (5) inducting capacity of ES. Inducting capacity refers to benefits provided by trees that support other benefits, but in which this relationship is not mutual. For example, all trees enhance the landscape, but only fruit trees provide food. In rural areas of Nysa, experts identified four criteria for DMs to consider in ranking ES: (1) inducting capacity, (2) the meaning of selected benefits delivered by trees for the local community, (3) the number of people using ES delivered by trees, and 4) the irreplaceability of selected ES. Consideration of these guidelines by experts during the second response to the questionnaire improved agreement between rankings. The final rankings of all 17 ES presented in Tables 2 and 3 show differences between rural and urban areas. We discuss the most important ones in detail.

**Table 2:** Ranking and consistency ratio (CR) of ES provided by trees in an urban area (Racibórz), described by local weights (LW) for individual ES and ES service classes. Rankings were obtained using AHP and calculated global weights (GW). CR of main service classes comparison: 0.02

Services class	LW	Individual ES	LW	GW	Rank
Habitat services (CR: 0.00)	0.438	Habitat and food source for animals	0.766	0.336	1
		Oxygen source	0.234	0.102	3
Provisioning services (CR: 0.00)	0.118	Supplying wood, branches, and leaves	0.165	0.019	11
		Delivery of fruits and nuts	0.835	0.099	4
Regulating services (CR: 0.04)	0.353	Regulating air humidity and soil moisture	0.308	0.109	2
		Sun protection (shadow)	0.151	0.053	7
		Positive impact on health and well-being	0.205	0.072	5
		Wind protection	0.039	0.014	12
		Protection against snowdrifts	0.038	0.013	13
		Noise reduction	0.063	0.022	10
		Air purification	0.196	0.069	6
Cultural services (CR: 004)	0.091	Educational usefulness	0.089	0.008	16
		Impact on the esthetics of space	0.355	0.032	8
		A sense of intimacy, separation from neighbors	0.117	0.011	14
		Place of recreation	0.267	0.024	9
		The tree as a witness to history: trees aged several hundred years, bearing traces of events, important for regional heritage	0.068	0.006	17
		Strengthening interpersonal bonds, psychological relationship between people and trees, sense of attachment to place (personal experience)	0.103	0.009	15

**Table 3:** Ranking and consistency ratio (CR) of ES provided by trees in a rural area (Nysa), described by local weights (LW) for individual ES and ES service classes. Rankings were obtained using AHP and calculated global weights (GW). CR of main service classes comparison: 0.014

Service class	LW	Individual ES	LW	GW	Rank
Habitat services (CR: 0.00)	0.218	Habitat and food source for animals	0.197	0.043	9
		Oxygen source	0.803	0.175	1
Provisioning services (CR: 0.00)	0.130	Supplying wood, branches, and leaves	0.679	0.088	4
		Delivery of fruits and nuts	0.321	0.042	10
Regulating services (CR: 0.01)	0.522	Regulating air humidity and soil moisture	0.106	0.055	7
		Sun protection (shadow)	0.149	0.078	6
		Positive impact on health and well-being	0.165	0.086	5
		Wind protection	0.095	0.050	8
		Protection against snowdrifts	0.043	0.022	13
		Noise reduction	0.192	0.100	3
		Air purification	0.250	0.131	2
Cultural services (CR: 004)	0.130	Educational usefulness	0.062	0.008	17
		Impact on the esthetics of space	0.231	0.030	12
		A sense of intimacy, separation from neighbors	0.241	0.031	11
		Place of recreation	0.241	0.031	11
		The tree as a witness to history: trees aged several hundred years, bearing traces of events, important for regional heritage	0.090	0.012	15
		Strengthening interpersonal bonds, psychological relationship between people and trees, sense of attachment to place (personal experience)	0.135	0.018	14

In the case of Racibórz, the ES receiving the highest weight in the final ranking was the provision of habitat and food resources for animals (Table 2). Experts indicated during discussions the importance of this benefit in urban areas, where animals (including insects, which are important to the ecosystem) have limited living space. The second most important ES was regulation of air humidity and soil moisture by trees. This ES mainly concerned water retention to help prevent flash flooding. The third ranked ES in the urban setting was the production of oxygen by trees, which experts stressed during discussion was an essential ecological function. However, the relatively small green spaces in Racibórz are not significant regionally or internationally as forest lands, which affected the local weight of this ES. Essential provisioning services, specifically providing fruit and nuts, ranked as the fourth most important ES in Racibórz. The most important CES in the urban area was the esthetics of space, but due to the comparatively low global weight of CES, this benefit placed eighth in the final ranking. Recreation ranked just after esthetics, but the weight of this ES was considerably lower.

In rural Nysa, the highest ranking ES was oxygen production. However, its weight was less than that given the highest ranked ES by experts in urban areas. Rural experts found air cleaning and noise reduction only slightly less important than oxygen production (Table 3). The ES rankings given to the provision of wood, branches and leaves indicated its importance in rural Nysa, but it was not among the top ten ES in urban Racibórz. It should be noted that the issue of providing leaves was not specifically mentioned as part of this ES during discussions, however neither was it raised as a disservice. Wind protection, similarly to provision of wood, branches and leaves, was likewise important in rural Nysa, but was not rated among the top ten ES by urban experts. In contrast, in Nysa, none of the cultural ES were among the top ten services, although two CES were among the top ten ES for urban Racibórz.

It is worth noting that regulatory benefits dominated the top ten ecosystem benefits provided by trees in both locations. Moreover, both urban and rural experts agreed that some of the 17 ES had low importance compared to other benefits. Low ranking ES included protection against snowdrifts, strengthening interpersonal bonds, and the tree as a witness to history.

## Discussion

The provision of different ES depends on tree size, age, species, and species mix (Aronson et al. 2017; Davies et al. 2017; Felton et al. 2020; Pretzsch et al. 2021). Without establishing objective criteria regarding which benefits are most important, enabling comparison of both environmental and social services, tree management and monitoring decisions may not address the greatest need, and determining how ES informs decision-making can be problematic. Our research addressed this problem by assessing the importance for local communities of 17 ES provided by trees in urban and rural areas and showed the methodology's usefulness for overcoming limitations observed in prior studies by using expert knowledge.

### ES ranking as a tool for increasing the effectiveness of tree protection strategies

In our study, urban and rural experts both placed the same eight ES among the top ten most important: source of oxygen, regulating air humidity and soil moisture, air purification, noise reduction, positive impact on health and well-being, delivery of fruits and nuts, sun protection, and supporting habitat for animals. If the importance of these eight ES is confirmed for other locations, it may mean these ES are universally important. Most of these ES belong to the regulating class of services and are among the most studied ES delivered by trees (Brockerhoff et al. 2017; Raum et al. 2019). In practice, it could suggest that the assessment of the most important ES provided by trees could be based only on objective information using remote sensing data. However, experts in the present study also valued some ES that in many other studies are often overlooked due to an absence of information about them or for other reasons, such as community perception of acceptability and risk. Ordóñez et al. (2019) state that municipal managers’ decision-making is rarely concerned with “urban forest success.” The absence of objective ES rankings can contribute to questionable decision-making by DMs such as taking pro-environmental actions that do not significantly address the most urgent needs of their community, but are socially well-regarded. For example, authorities should not focus on distributing trees for planting to private landowners if the city's most pressing need is to avoid heat islands in the city center, because private land is more often found on the city's outskirts. The strategy for implementing such planting decisions should instead be determined by objectively established goals, which can be assessed using, for example, the priority protection index (Lin 2020). If environmental progress were evaluated based on the ranking of ES provided by trees, then achieving success or failure could be measured taking ES into account, which is likely to provide a more reliable assessment of activities to protect and promote urban forests.

The results of this study indicate the value that municipalities can obtain by ranking ES provided by trees. This value is related to better understanding of local problems concerning different categories of ES and their relative importance (such as accessibility to green areas providing CES or environmental pollution).

Moreover, DMs may verify the meaning for local communities of those ES whose assessment and management require more complex data sources than remote sensing. Establishing the hierarchy of ES for a municipality will demonstrate key issues related to tree management, which can be prioritized so that they receive appropriate action. The need for communities to conduct ES rankings is shown by the difference in prioritizations obtained between nearby urban and rural areas in this study and the different criteria for evaluating ES that experts worked out during group discussions. However, while local context can lead to unique rankings of ES, the results of this study indicate that some ES may be universally important for specific types of municipalities.

For Racibórz and presumably other urban areas, the most important ES was the provision of habitat and food resources for animals. This benefit is directly influenced by biodiversity and was similarly determined to be the most critical ES in South Korea urban national and neighborhood parks (Jang-Hwan et al. 2020). This service depends not only on the presence of trees but also on how they are managed. Good practices to provide food and habitat for wildlife include planting native tree species and creating heterogeneous urban green spaces, both on public and private land (Aronson et al., 2017). Without these management steps, an increase in urban tree cover may not necessarily increase biodiversity since not all types of urban green spaces provide good animal habitat, i.e., impermeable ground under trees significantly reduces its habitat potential. It should be emphasized that tree habitat and food resources for animals is an “inductive” ES, which was a high priority according to both urban and rural experts in this study. Usually, when this ES is provided, other ES will also be delivered, since there is a positive relationship between biodiversity and most ES (Brockerhoff et al. 2017).

Brockerhoff et al. (2017) showed in their literature review that biomass productivity increases with tree species richness, which influences the supply of both provisioning and regulating ES. Thus, variation in tree age and species is conducive to creating a habitat for various species of animals, but may also support, for instance, the resilience of forest trees to wind. Only in the case of CES is the relationship between habitat provided by trees and access to cultural benefits ambiguous. On the one hand, greater biodiversity among trees enhances cultural values, such as education and esthetics. On the other hand, dense afforestation can inhibit people who would like to use forest areas for recreation. As a result, Brockerhoff et al. (2017) underline that relation between CES and biodiversity requires further research. In most cases, however, we conclude that supporting the habitat properties of trees favors creating spaces that allow access to multiple ES.

ES rankings in other urban municipalities are needed to determine whether the high ranking of the animal habitat ES in this study applies elsewhere. If the high priority of this ES is confirmed in other locations, it would indicate the need for better tree protection efforts in cities. This would require urban management practices that protect and promote biodiversity, instead of a less focused effort that only looks to increase tree cover. Promoting biodiversity is especially important in situations where this ES may be undervalued (Potgieter et al. 2018). In addition, our study supports the need identified by Brockerhoff et al. (2017) for additional research to help dispel uncertainty about the relationship between biodiversity and the availability of CES.

Our study shows that CES significantly differentiated urban and rural areas, as ES from this class appeared among the top ten priorities only in Racibórz. This supports the results of Ali et al. (2020), who investigated the willingness of people in urban and rural areas to pay for ES (specifically in their case, ES delivered by rivers in the Hexi Corridor Region in China). In rural areas, creating recreational opportunities (the only CES included by Ali et al. (2020)) was assessed by residents as the least important reason for incurring additional costs. Differences in the perception of ES (and disservices) between rural and urban areas are important in the development of local public policies regarding the management of trees, and more broadly, green spaces (Rodríguez-Morales et al. 2020). Taking these differences into account will better reflect the public demand on ES in urban and rural areas, and can be expected to increase public acceptance of particular management actions (Drillet et al. 2020).

In rural areas in our study, a greater priority was given to ES related to providing wood and branches, and to wind protection, which both ranked in the top ten most crucial ES. While providing wood and branches is promoted by forest management carried out by other levels of public administration beyond the municipal level in Poland, providing wind protection requires appropriate decision-making by local authorities. To support wind protection, DMs should preserve trees along roads and fields, and promote new plantings in these locations (Davies et al. 2017), which is also essential for enhancing the European Greenway corridors network that struggles to gain support in rural areas. Carlier and Moran (2019) describe a noticeable decline in such corridors in European agricultural landscapes, even though the corridors provide other significant benefits, such as noise reduction and air pollution abatement that were highly valued by rural experts in this study. ES evaluations could counter this worrying trend, by showing the exceptional importance of roadside trees in the countryside. This, in turn, can be used to obtain support for management activities that ensure the benefits trees provide to rural communities are delivered.

It should be remembered that geographic, location and taxonomic biases make generalizations about the ranking of ES difficult (Shwartz et al. 2014). These make developing universal guidelines for tree protection challenging and it requires further investigation. There may also be a need to consider ES in landscapes beyond urban and rural, such as in rapidly expanding areas of peri-urban development. In this regard, Spyra et al. (2021) emphasized the need to consider ES provided by open spaces in peri-urban locations in regional governance documents. Thus developing ES rankings for trees in transition areas between towns and cities may be crucial for creating effective protection strategies for them.

### Procedure pros and cons

The procedure used in this study is a new approach that avoids some of the limitations of prior methods of rankings ES. First, by applying the AHP method using a hierarchical structure we were able to compare very different ES delivered by trees—much more so than has been done in previous research. Furthermore, combining this approach with discussions in mini focus groups significantly improved the levels of agreement among experts. Reducing the number of comparisons during discussion sessions to the seven most conflicting ones allowed for much better agreement among experts when they reassessed their rankings, especially for experts from the urban area. This indicates that it is unnecessary to discuss all possible ES comparisons, as is usual for group weighting using the AHP method. Instead, it was sufficient to create general guidelines based on selected ES, which experts then considered when filling out questionnaires individually. The procedure we used enabled us to take advantage of both AHP approaches (with and without discussion), reducing their negative consequences.

It should be noted that unequal distribution of ES in pairwise comparisons can affect the results of ES rankings. This effect could be eliminated by modifying local weights of the main ES classes based on the number of ES present in each class, rather than simply using their relative importance. This approach can be justified when experts agree, upon completing the questionnaire for the first time, that ecosystem service classes are equally important. This did not happen in the present study, since experts representing both urban and rural areas identified the regulating and habitat service classes as being of much greater importance than other classes. In this situation, the solution was to make experts aware of the consequences of the hierarchical structure approach and to instruct them on how to select appropriate weights for the main ES classes, in order to limit the effects of unequal distribution of ES. When this was done in our study, the change in local weights assigned to the main ES classes after discussion sessions showed that experts took into account the number of ES within classes, although this was not a crucial criterion for them.

## Conclusion

The ES concept offers a comprehensive classification that describes the range of benefits provided by trees. However, ES cannot all be maximized at the same time, and therefore methods are needed to prioritize management actions. Our research shows that nature conservation strategies and policies should consider the relative local importance of ES provided by trees for communities. We suggest that AHP-based ES rankings offer important insights for tree protection planning. We argue that this approach can be instrumental in resolving trade-offs for DMs. The procedure proposed in this paper overcomes many of the limitations present in previous studies and could be a useful approach in future research concerning both – general recommendations concerning ES and site-specific ES ranking.

However, because of the complexity of ES studies, we recommend developing general guidelines for assessment and conservation of ES provided by trees in urban and rural areas. This could be a starting point for future research and help prioritize objectives for tree protection. Our results indicate that eight ES may have universal relevance independent of location. There are: (1) oxygen supply, (2) air humidity and soil moisture regulation, (3) air purification, (4) noise reduction, (5) positive impact on health and well-being, (6) delivery of fruits and nuts, (7) sun protection, and (8) supporting habitat for animals. Most of these ES are regulating benefits, which are already monitored quite often, but there are also some that have not received enough attention so far. One of them, the provision of animal habitat and food, turned out to be essential but often overlooked in urban areas, and requires attention from policy makers. In practice, providing animal habitat ES should be crucial in designing urban green spaces. Our results also show the particular importance of CES in cities, although these services should not be the deciding factor in planning urban greenery spaces. We also indicate the critical role that trees play in rural areas in providing wood and branches, and the importance of roadside trees in reducing noise, pollution and protecting against wind, which are particularly important in the countryside.

Approaches for developing tree protection strategies, including accounting for differences in ES delivered by trees in urban and rural areas, require further investigation. Identifying key priorities for tree-related environmental management needs to address the specific characteristics of the areas under consideration. While the urban–rural divide in ES can be part of the basis for setting management priorities for regional and local DMs, it should not be the only considered issue since other area types may also require special attention in this regard.

## Supplementary Information

Below is the link to the electronic supplementary material.

Supplementary file1 (PDF 99 kb)

## Footnotes

1 Statistics of Poland, Local Data Bank—https://bdl.stat.gov.pl/BDL/start (Accessed on 28–04-2018).
