# Correction to: Ranking ecosystem services delivered by trees in urban and rural areas

**DOI:** 10.1007/s13280-022-01747-7

**Published:** 2022-05-11

**Authors:** Przewoźna Patrycja, Mączka Krzysztof, Mielewczyk Marcin, Inglot Adam, Matczak Piotr

**Affiliations:** 1 Faculty of Geographical and Geological Sciences, Adam Mickiewicz University, Bogumiła Krygowskiego 10, 61-680 Poznań, Poland; 2 Faculty of Sociology, Adam Mickiewicz University in Poznań, ul. Szamarzewskiego 89c, 60-568 Poznań, Poland; 3 Institute of Anthropology and Ethnology, Faculty of Anthropology and Cultural Science, Adam Mickiewicz University in Poznań, ul. Szamarzewskiego 89, 60-568 Poznań, Poland; 4 Faculty of Civil and Environmental Engineering, Gdańsk University of Technology, ul. Narutowicza 11/12, 80-233 Gdansk, Poland

## Correction to: Ambio 10.1007/s13280-022-01722-2

The article “Ranking ecosystem services delivered by trees in urban and rural areas”, written by Przewoźna Patrycja, Mączka Krzysztof, Mielewczyk Marcin, Inglot Adam and Matczak Piotr, was originally published electronically on the publisher’s internet portal on 28 March 2022 without open access. With the author(s)’ decision to opt for Open Choice the copyright of the article changed on 02 May 2022 to © The Author(s) 2022 and the article is forthwith distributed under a Creative Commons Attribution 4.0 International License, which permits use, sharing, adaptation, distribution and reproduction in any medium or format, as long as you give appropriate credit to the original author(s) and the source, provide a link to the Creative Commons licence, and indicate if changes were made. The images or other third party material in this article are included in the article’s Creative Commons licence, unless indicated otherwise in a credit line to the material. If material is not included in the article’s Creative Commons licence and your intended use is not permitted by statutory regulation or exceeds the permitted use, you will need to obtain permission directly from the copyright holder. To view a copy of this licence, visit http://creativecommons.org/licenses/by/4.0.

The original article has been corrected.

