# Supplementary material for: Ranking ecosystem services delivered by trees in urban and rural areas
Source: Ambio. 2022 Mar 28;51(9):2043–57. doi: 10.1007/s13280-022-01722-2 (PMC9287513; doi:10.1007/s13280-022-01722-2)
Supplement: Supplementary file 1 — Supplementary file1 (PDF 99 kb) [file 13280_2022_1722_MOESM1_ESM.pdf]

Ambio

Electronic Supplementary Material

This supplementary material has not been peer reviewed.

Title: **Ranking ecosystem services delivered by trees in urban and rural areas**

Authors: Przewoźna P., Mączka K., Mielewczyk M., Inglot A., Matczak P.

Appendix S1. Degree of consent on which of the compared ES is more relevant

a) Scale representing degree of consent between experts:

degree of consent	category	Meaning
1	full agreement	all experts have chosen the same ES as more important (or they agree that the ES are equally important)
2	not full agreement	four experts have chosen the same ES as more important and one could not make the decision or four experts agree that two ES are equally important and one chooses one ES as more important
3		three experts have chosen the same ES as more important and two could not make a decision
4		two experts have chosen the same ES as more important and three could not make a decision
5	indecisiveness	two experts have chosen the opposite ES as more important and three could not make a decision
6	lack of agreement	four experts have chosen the same ES as more important and one made the opposite decision
7		three experts have chosen the same ES as more important, one made the opposite decision and one could not make a decision
8		two experts have chosen the same ES as more important and one made the opposite decision, two could not make a decision
9	conflict	three experts have chosen the same ES as more important and two made the opposite decision
10		two experts have chosen the same ES as more important and two made the opposite decision, one could not make a decision

b) The most conflicting pairs of ES within each comparison group **[degree of consent]**, chosen for discussion in mini focus groups in Racibórz:

1. Cultural & amenity VS provisioning **[9]**,
2. A sense of intimacy, separation from neighbors VS educational usefulness **[7]**,
3. Strengthening interpersonal bonds VS the tree as a witness to history **[9]**,
4. Wind protection VS positive impact on health and well-being **[9]**,
5. Air purification VS regulating air humidity and soil moisture **[10]**,
6. Habitat and source of food for animals VS oxygen source **[7]**,
7. Delivery of fruit and nuts VS supplying wood, branches, and leaves **[3]**,

and in Nysa:

1 Cultural & amenity vs provisioning **[10]**,

2 Delivery of fruit and nuts VS supplying wood, branches, and leaves **[10]**,

3 Place of recreation VS impact on the aesthetics of space **[9]**,

4 Strengthening interpersonal bonds VS impact on the aesthetics of space **[10]**,

5 Positive impact on health and well-being VS sun protection (shade) **[10]**,

6 Noise reduction VS sun protection (shade) **[9]**,

7 Habitat and food source for animals VS oxygen source **[8]**

c) Share of pairs representing compliance level before (1) and after discussion (2) in each case study area in a ten-degree of consent scale. Green and yellow represent pairs with lower or higher agreement (1-5), red colors are pairs with- a lack of agreement (6-10).

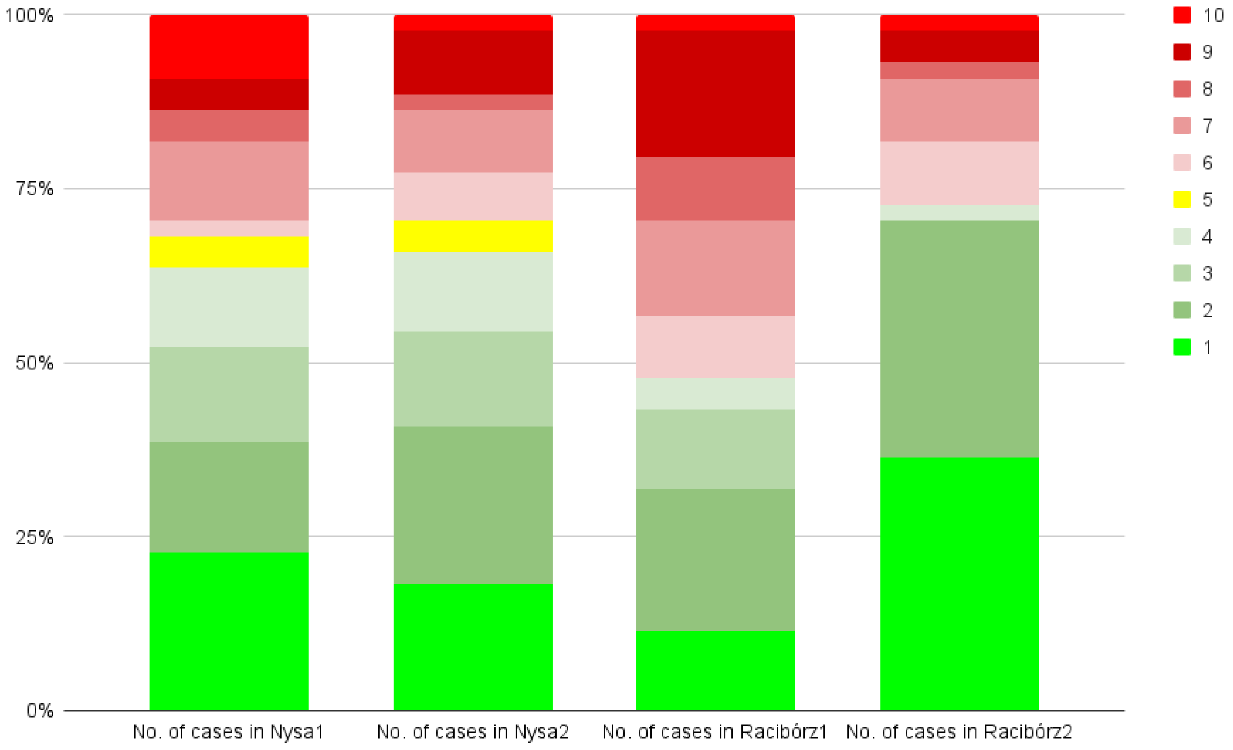

Appendix S2 – Mini Focus Group

We applied mini focus group discussion between two AHP measurements, which is a qualitative, exploratory research method. Typically, a focus group brings together eight to ten qualified people for a face-to-face discussion of a particular topic. In bigger groups, interactions among the participants are less effective, and discussions can be hard to control (Edmunds 1999). However, in our research we applied a mini focus group (Edmunds 1999; Greenbaum 1998) which slightly differed from a standard focus group. Mini focus groups include from 4 to 6 participants and there is more emphasis on the topic and less on polling the participants, which is especially relevant when participants are experts with higher levels of knowledge on investigated topics. Therefore, it gave us greater observational opportunities and more chances to do hands-on testing than in larger groups. For each mini group we also did over-recruiting, as generally 8 or 9 qualified contacts should be recruited to guarantee a group size of 4 or 6 participants. Finally, we obtained two groups composed of 5 participants each.

Both mini focus group discussions lasted 4 hours each and were carried out using the same scenario. The scenario was based on the Utrecht method (Bolt et. al 2015; Shawahna 2018). The Utrecht method is a discussion tool that focuses on daily life dilemmas of professionals. It was developed for healthcare providers facing moral deliberations, often starting with action-guiding questions such as “what should I do?” and ends with a concrete decision after comparison of possible solutions. On the one hand, compared to other discussion tools as citizen jury (Veasey 2004), the world cafe (Carson 2011), deliberative polling (Fishkin 2018), etc., the Utrecht method allows relatively small groups of people to be involved, discussing a precise and feasible number of questions, building a fairly simple structure, and has been already combined with AHP in previous research (Shawahna 2018). On the other hand, similarly to the discussion tools mentioned, the Utrecht method enables us to reflect reasons to justify decision that are made, taking into account normative perspectives that various groups of stakeholders hold in practice.

The Utrecht method is guided by 8 questions focused on the professional and/or ethical issue in the discussed case, potential decisions which might be taken, comprehensiveness of relevant information, potential risks, perspectives of various stakeholders, and implementation of preferred action (please see original question list in Bolt et al. 2015). In our research, we adapted the original list of questions after pilot testing within the simulation of discussion in a 5 person research team.

Duration	Meeting point
20’	Welcoming the participants<br>Presentation of the purpose and formula of the meeting<br>Familiarization round. Each expert introduces himself:<br>name<br>education<br>current occupation
20’	Presentation of results of initial measure of AHP and identification of ecosystem services comparisons with the highest rate of noncompliance for discussion.
50’	**First part of the discussion (comparing 2-4 pairs depending on time consumption)**<br><br>**Example:** Impact on aesthetics of space vs. educational usefulness<br><br>Each pair compared will be discussed based on the following questions:<br>How do you define the compared pairs of benefits provided by trees? Do you use them and how?<br>From your perspective, which benefit is more important? What is most important about these benefits and what is less important or unimportant? Let's focus primarily on the context of Nysa/Raciborz.<br>What do you see as the potential benefits (potential gains) from considering one benefit more important than the other? I remind you that we are particularly interested in the context of your municipality.<br>What do you see as the potential downsides (risks, costs) of considering one benefit as more important than the other? I remind you that we are particularly interested in the context of your municipality.<br>For which groups of inhabitants (residents, schools, entrepreneurs, or other key groups of so-called stakeholders) are these benefits the most important?<br>Let us now collect the most important arguments for and against considering a given benefit as more important.

	At the end of the discussion, I will ask each of you to briefly answer the questions: which benefit would you currently consider more important (in light of what was said in the discussion)?
10’	**Short break**
30’	**Second part of the discussion (remaining pairs)**<br>After completing the discussion on all the pairs compared, ask the question:<br>How can we give a benefit more weight in practice?
30’	**Long break + time to prepare a summary of the meeting**
20’	Meeting summary and conclusions

Supplementary references:

Bolt, I., van den Hoven, M., Blom, L., & Bouvy, M. (2015). To dispense or not to dispense? Ethical case decision-making in pharmacy practice. International journal of clinical pharmacy, 37(6), 978-981.

Carson, L. 2011. Designing a public conversation using the World Cafe method:[Paper in themed section: The Value of Techniques. Martin, Brian (ed.).]. Social alternatives, 30(1), 10-14.

Edmunds, H. (1999). The focus group research handbook. The Bottom Line.

Fishkin, J. S. 2018. Deliberative polling. In The Oxford handbook of deliberative democracy. Oxford University Press 314-328.

Greenbaum, T. L. (1998). The handbook for focus group research. Sage.

Shawahna, R. (2018). Combining and using the utrecht method and the analytic hierarchy process to facilitate professional and ethical deliberation and decision making in complementary and alternative medicine: a case study among a panel of stakeholders. Evidence-Based Complementary and Alternative Medicine, 2018.

Veasey 2004, K. Citizen jury handbook, Jefferson Centre.
